# The Traffic Light Planning Algorithm for Breast Augmentation Mastopexy: A Case Series Analysis on Optimizing Early Ptosis

**DOI:** 10.7759/cureus.94758

**Published:** 2025-10-16

**Authors:** Amit Nijran, Stephen Ali, Mohamed Maklad

**Affiliations:** 1 Plastic Surgery, Queen Elizabeth Hospital Birmingham, Birmingham, GBR; 2 Reconstructive Surgery/Regenerative Medicine Research Centre, Swansea University, Swansea, GBR; 3 Plastic Surgery, Enhance Medical Group, Birmingham, GBR

**Keywords:** aesthetic surgeries, augmentation mastopexy, breast plastic surgery, planning algorithm, plastic and reconstructive surgery

## Abstract

Background

Breast augmentation is one of the most commonly performed aesthetic procedures, yet cases involving breast ptosis often require a more individualized approach. Determining the optimal strategy for combining augmentation with lift procedures can be challenging, particularly for surgeons early in their aesthetic practice. This study introduces the Traffic Light Planning Algorithm as a structured and objective framework to assess breast anatomy, guide implant positioning, and determine the need for adjunctive lift procedures. The algorithm categorizes patients into three groups (green, amber, and red) to help select the most appropriate management plan. This paper focuses on the amber zone, representing cases with early ptosis, which often present the greatest challenge in achieving optimal correction.

Objectives

The primary aim of this study was to evaluate the safety and patient satisfaction outcomes associated with the Traffic Light Planning Algorithm over five years (2018-2023). The secondary objective was to conduct a focused retrospective case series analysis of the “amber zone” over 12 months (December 2022-December 2023). This subset analysis aimed to assess whether Mentor MemoryGel Xtra implants (Mentor Worldwide LLC, Irvine, CA, USA) could correct early ptosis without adjunctive mastopexy and to provide supporting evidence for the validity of the algorithm. A representative case report is also included to illustrate the practical application of this approach.

Methods

A comprehensive description of the algorithm is provided. Over the five-year study period, patient satisfaction, complication rates, and revision rates were analyzed. The 12-month subset analysis further explored patient satisfaction and various patient and surgical factors through cross-tabulation. This retrospective study design was appropriate for assessing real-world outcomes related to patient satisfaction and complications associated with this new structured planning algorithm. The Likert scale was used as an effective tool for evaluating patient satisfaction.

Results

During the study period, 9,000 breast consultations and 3,000 implant surgeries were performed by the senior author (MM). Overall, 93% of patients reported high satisfaction with their outcomes, with low revision rates of 2% for primary augmentations and 6% for augmentation mastopexies. This method proved particularly effective for cases of early ptosis, providing a structured and reproducible decision-making framework. The subset case series of 59 patients over a 12-month period showed high satisfaction levels: 54 (91.5%) were “very happy,” 3 (5.1%) were “happy,” 1 (1.7%) was “satisfied,” and 1 (1.7%) was “not happy.” Complications were reported in four cases (6.8%), including bilateral bottoming out, two cases of implant loss due to infection, and one case of unilateral capsular contracture. The remaining 55 patients (93.2%) experienced no postoperative complications.

Conclusions

The algorithm provides a reliable and objective framework for planning breast augmentation, particularly valuable for surgeons new to aesthetic practice. By promoting personalized, tissue-based surgical planning, this approach helps minimize complications and enhance patient satisfaction. The case series further demonstrates that applying the algorithm, together with the use of Mentor MemoryGel Xtra implants, can offer an effective strategy for avoiding mastopexy in appropriately selected amber zone patients.

## Introduction

Breast augmentation is one of the most commonly performed aesthetic procedures worldwide [1,2]. While implants alone can yield satisfactory results in patients without significant breast ptosis, or early ptosis, cases involving varying degrees of ptosis present additional challenges. For surgeons, particularly those new to aesthetic practice, determining when and how to combine breast augmentation with mastopexy can be daunting. Common pitfalls include underestimating the degree of ptosis, selecting an inappropriate implant or surgical approach, and failing to adequately address asymmetries.

Over the years, several classification systems and decision-making frameworks have been described to guide management in augmentation-mastopexy [3-7]. Traditionally, the Regnault classification, introduced in 1976, has served as a foundation for categorizing ptosis based on the position of the nipple-areola complex (NAC) relative to the inframammary fold (IMF) [3]. However, as surgical methodologies have advanced, the limitations of a solely positional classification have become more apparent, especially for cases of mild, early ptosis. Modern approaches emphasize a shift toward tissue-based assessments, which allow for a more individualized plan by factoring in unique tissue characteristics and elasticity alongside NAC positioning. However, inexperienced surgeons may find these methods complex, and inconsistencies in defining landmarks can lead to subjective assessments and unpredictable results.

To address these challenges, we developed the “Traffic Light Planning Algorithm.” This algorithm uses two key anatomical references to streamline decision-making: the IMF and the expected new nipple position raised by the implant as identified by the Per Hedén Manoeuvre (patients lifting their arms above their head while in a relaxed standing position) [8]. By providing a clear, objective system for categorizing breast ptosis and guiding the choice of implant, position, and mastopexy technique, the algorithm helps surgeons navigate the complexities of augmentation-mastopexy planning. The implant-created nipple-lift concept, as introduced by Hedén [8], is central to this framework, serving as a fixed reference point that aids in accurately assessing the vertical excess (VE) and determining the need for adjunctive lift procedures.

In this article, we describe and analyze the Traffic Light Planning Algorithm in detail, explain the marking technique, and demonstrate how it can guide surgical decisions. This structured approach can reduce subjectivity, streamline planning, and help new surgeons build confidence in managing a wide spectrum of breast ptosis presentations. We also present a case series analysis applying this algorithm to the most challenging group, the amber zone of the Traffic Light Planning Algorithm, using Mentor MemoryGel Xtra implants (Mentor Worldwide LLC, Irvine, CA, USA). These implants provide a subtle built-in support that can often eliminate the need for a mastopexy.

## Materials and methods

**Figure 1:**
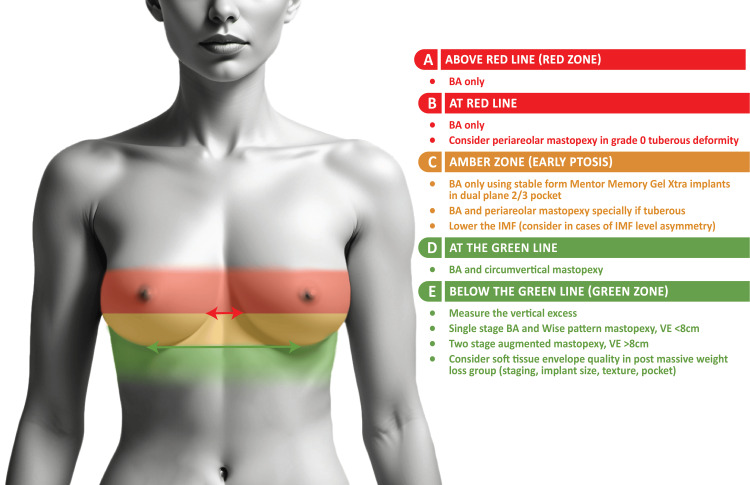
Traffic Light Planning Algorithm Image Credit: Stephen Ali.

Figure 1 presents the key reference lines of the Traffic Light Planning Algorithm.

Green line: inframammary fold

The first step is to identify and mark the IMF, which serves as the “green line.” The IMF defines the lower boundary for implant positioning and provides a stable landmark against which the surgeon can assess nipple position and vertical skin excess. It is not uncommon to have IMF asymmetry. In this case, each fold is individually marked.

Red line: using the Per Hedén Manoeuvre

The Per Hedén Manoeuvre is performed with the patient standing in a relaxed anatomical position [4]. Starting at the IMF, the surgeon gently follows the natural contour of the lower breast pole upward, marking a point approximately 2-3 cm above the IMF to represent the implant-created expected new nipple position [8]. This position can be confirmed by asking the patient to place their hands above their head and assessing the new nipple position. This red line serves as a crucial reference for evaluating ptosis. By establishing a consistent anatomical apex, the surgeon can more objectively determine whether adjunctive mastopexy is needed.

Applying the Traffic Light Planning Algorithm

With the green (IMF) and red (apex) lines marked, the existing nipple position is evaluated relative to these lines. The “zones” guide surgical decisions.

Nipple Above the Red Line

If the nipple is located above the red line, this indicates no ptosis. Breast augmentation alone with an appropriate implant choice (e.g., a smooth or slightly overfilled form-stable silicone gel implant for upper pole support) is generally sufficient.

Nipple at the Red Line

If the nipple aligns with the apex line, there is mild or borderline ptosis. In most cases, augmentation alone may suffice, although a peri-areolar mastopexy or intra-areolar mastopexy could be considered for mild tuberous deformities or areolar puffiness.

Nipple Between the Red and Green Lines (Early Ptosis, Amber Zone)

When the nipple sits below the apex but above the IMF (the so-called amber zone), subtle ptosis is present. Overfilled silicone gel implants can provide a slight internal lift. If there is Regnault Grade I ptosis or a tuberous deformity, a peri-areolar mastopexy may be added. Adjustments to the IMF position can be made if asymmetry is present.

Nipple at the Green Line

A nipple at the green line usually represents more advanced ptosis. In these cases, combining augmentation with a circumvertical (lollipop) mastopexy can provide adequate lift and reshaping. However, caution is advised if the nipple is just at the IMF without VE, as some patients may not require a full circumvertical mastopexy, and a less invasive lift could suffice depending on individual tissue characteristics.

Nipple Below the Green Line (Significant Ptosis, Green Zone)

When the nipple falls below the IMF, VE must be measured. For VE < 10 cm, a single-stage augmentation-mastopexy using a Wise pattern incision is typically effective. For VE > 10 cm, often the case in massive weight loss patients or significant laxity, a two-stage approach (mastopexy first, followed by augmentation after healing) may be considered to minimize complications such as bottoming out or poor shape retention.

Study design

Using the Traffic Light Planning Algorithm to guide management, a retrospective case series analysis was conducted to evaluate the outcomes of breast augmentation with Mentor MemoryGel Xtra implants in patients with early ptosis, within the amber zone of the algorithm. This approach provides a real-world assessment of the algorithm’s effectiveness in clinical practice. The focus on this subgroup reflects its complexity, given the wide variation in implant types, profiles, and heights. The study included patients who underwent surgery between December 2022 and December 2023, all performed by a single surgeon (MM), with a minimum follow-up period of 12 months required for inclusion. The primary outcome was patient satisfaction, while secondary outcomes included complication rates and revision rates.

Surgical planning

For patients with early ptosis, the surgical approach was determined using a tissue-based assessment model, which offers a nuanced alternative to the traditional Regnault classification [3]. While Regnault’s system indicates that patients with Grade II ptosis generally require an uplift, the tissue-based assessment allows for tailored decisions based on specific laxity factors. In particular, patients presenting with Grade I or II ptosis but exhibiting only moderate laxity may be managed with augmentation alone, without the need for a lift.

Unlike traditional MemoryGel implants, which are typically underfilled to create a softer, more malleable feel, MemoryGel Xtra implants are slightly overfilled to the high end of the allowable volume, which creates a scalloped edge and promotes a firmer central projection [9]. The scalloped edges create a peripheral pillar-like effect, effectively offering an “internal lift” suited to cases of mild ptosis. Additionally, pairing high-profile, textured implants with a dual-plane 2 or 3 (DP2 or DP3) pocket allows for enhanced stability, reducing further ptosis risks and supporting the soft tissue envelope. The advantage of this unique implant shape precludes the use of particularly large-sized implants to fill the skin envelope. Therefore, the longevity of the result is longer than when normal, larger-sized implants are used.

Historically, surgeons tended to correct early ptosis by placing the implant in the sub-glandular plane, assuming that filling the loose skin would suffice to provide the desired lift. Although this often produced satisfactory results initially, patients frequently experienced early bottoming out due to the moderate skin elasticity and the weight of the implant. This often necessitated corrective mastopexy soon after the primary surgery. In our study, implants were inserted in a dual-plane approach, as this balances the risk of bottoming out and waterfall deformity effectively. Patients with poor soft tissue envelopes are at risk of waterfall deformity if implants are placed in DP1. On balance, placing the implants in DP2 or DP3, essentially half sub-muscular and half sub-glandular, ensures that the lower portion of the implant helps fill the stretched or excess skin while the upper portion, in contact with the muscle, remains well-supported and stable, providing a better long-term result.

Comprehensive patient assessment is essential when selecting the optimal approach for managing early ptosis. Evaluation begins with visual inspection and measurement, including NAC positioning within the amber zone, as defined by the Traffic Light Planning Algorithm. Tissue-based assessment is the next key step. Anterior pull skin stretch (APSS) and nipple-to-IMF (N-IMF) distance under maximal stretch are measured to evaluate soft tissue envelope characteristics. Patients are considered suitable candidates for this surgical option when moderate laxity is confirmed, defined as APSS < 4 cm and N-IMF under maximum stretch < 10 cm. Measurements exceeding these thresholds indicate reduced tissue elasticity, for which a form of mastopexy is recommended. Finally, breast base width (BBW) is measured to ensure appropriate implant base selection.

Implant selection is then tailored to the patient’s anatomy. For early ptosis, high-profile, micro-textured implants are preferred, as their “velcro-like” texture against the muscle provides extra stability and maintains the upper pole fullness, and their high profile enhances projection, lifting the low nipple position. MemoryGel Xtra implants, with optimized shell-gel filling, align with these goals by adding volume precisely where needed and reinforcing the soft tissue envelope. In patients with moderate laxity, dual-plane pocket placement (DP2 or DP3) is used. By increasing the sub-glandular component of the pocket, the incidence of the waterfall effect is very low. Precise planning and execution of the level of parenchyma-muscle separation are crucial, as over-separation of the parenchyma from the muscle can increase the risk of the implant bottoming out.

For cases of early ptosis, implants sized to BBW + 50-60 cc often accommodate moderate laxity, striking a balance between projection and skin tension. Once chosen, the implant’s lower pole arc should match the N-IMF distance under maximal stretch. Once the size is decided, this should correspond to the patient’s goal. The implant profile can be changed to Moderate Plus if this would match the patient’s goal better.

The recent GLOW Clinical Trial on Mentor MemoryGel Xtra breast implants demonstrated promising three-year safety and effectiveness outcomes across 287 participants in primary and revisional breast augmentation and reconstruction [9]. However, while these findings underscore MemoryGel Xtra’s effectiveness in standard augmentation and reconstruction, no studies have yet examined its role specifically in addressing early ptosis, which is addressed in our study.

The suitability of MemoryGel Xtra implants for these cases was initially indicated if the NAC fell within the amber zone of the Traffic Light Planning Algorithm. However, to confirm candidacy for implant-only management, further tissue-based assessments were performed. This included ensuring the APSS was less than 4 cm, and the nipple-to-IMF distance measured between 10 cm under maximum stretch. If these conditions were met, the patient could achieve satisfactory outcomes with augmentation alone, effectively addressing early ptosis even in cases where Regnault’s classification might traditionally suggest an uplift.

An IMF incision approach is used for access in all cases. The planning dual-plane placement is guided by the position of the red line of the Traffic Light Planning Algorithm. Patients with the nipple positioned at or above the red line typically underwent DP1 placement, while those with the nipple below this reference line were more suited to DP2 or DP3, depending on the specific anatomical requirements. Intra-operative adjustments allowed further refinement of the pocket plane, maximizing both aesthetic outcomes and patient satisfaction.

Outcome measures

Data were collected on patient demographics (age, BMI, smoking status, and pregnancy history), implant characteristics (size and profile type), and surgical approach (pocket placement type). Implant size was recorded as the numerical value indicated by the implant model, and profile type was categorized as either “THP” or “TM+.” The type of dual-plane pocket used during surgery (DP1, DP2, DP3, or other) was also documented. The primary outcome measure was patient satisfaction, assessed at follow-up visits using a five-point Likert scale [10], where patients rated their satisfaction as “very dissatisfied,” “dissatisfied,” “neutral,” “satisfied,” or “very satisfied.” Additionally, the BREAST-Q questionnaire [11] was administered preoperatively and postoperatively to evaluate satisfaction and quality of life. Secondary outcome measures included the incidence of complications.

Statistical analysis

Descriptive statistics were generated to summarize patient characteristics, implant sizes, pocket placements, and outcomes. Categorical data were summarized as frequencies and percentages. Continuous variables, including age and BMI, were described using means and standard deviations. Cross-tabulations were conducted to explore relationships between satisfaction levels and factors like smoking status, pregnancy history, implant size, and pocket type. Group comparisons were performed to calculate mean satisfaction scores across different groups. Statistical analysis was performed using RStudio (R Core Team, R Foundation for Statistical Computing, Vienna, Austria) [12].

Ethics statement

This study was conducted in accordance with the principles of the Declaration of Helsinki. Institutional review board approval was deemed not required, as this is a descriptive original technique article and case series analysis of retrospective records. Informed written consent was obtained from the patients for the open-access publication of this manuscript.

## Results

During the five-year period (2018-2023), the Traffic Light Planning Algorithm was applied in over 9,000 breast consultations and 3,000 implant surgeries. Among these patients, 93% (2,790/3,000) reported high satisfaction with their aesthetic outcomes at 12 months or longer, as measured by standardized patient-reported outcome tools (Likert scale or BREAST-Q). The revision rate was 2% (60/3,000) for primary augmentations and 6% (180/3,000) for augmentation mastopexies. Revisions primarily addressed minor asymmetry, capsular contracture, or residual ptosis. No serious complications were directly attributed to the use of the algorithm.

Between December 2022 and December 2023, 59 patients were classified within the amber zone, demonstrating signs of early ptosis as defined by the algorithm. All underwent augmentation alone using Mentor MemoryGel Xtra implants.

Patient demographics and surgical characteristics of the subgroup analysis

In this subgroup analysis, the mean patient age was 32.3 years (SD = 8.32), and the mean BMI was 22.2 (SD = 2.69). Among the patients, 21 (35.0%) were current smokers, and 76.7% had a history of at least one pregnancy. Implant sizes ranged from 255 cc to 515 cc, with a mean size of 365 cc. Most implants (78%) were THP profile, while the remaining 22% were TM+. Pocket placements included DP1 (25%), DP2 (50%), and DP3 (25%), with DP2 being the most commonly used approach.

**Table 1:** Patient demographics and implant characteristics

Patient/implant characteristics	Mean	Standard deviation/range
Age	32.3	8.32
BMI	22.2	2.69
Smoker	21 (35%)	-
>1 pregnancy	45 (76.7%)	-
Implant sizes	365 cc	255-515 cc

Patient satisfaction

All 59 patients in the subgroup analysis provided satisfaction responses: 54 (91.5%) were “very happy,” 3 (5.1%) were “happy,” 1 (1.7%) was “satisfied,” and 1 (1.7%) was “not happy” due to implant size. Relationships between satisfaction and various patient and surgical factors were explored through cross-tabulation analyses.

Comparable satisfaction levels were observed among smokers and non-smokers, with the majority in both groups reporting being “very happy” with their outcomes. Interestingly, all patients who reported being “happy” or “satisfied” were non-smokers, while the only “not happy” response came from a smoker, suggesting that smoking status did not have a significant effect on overall satisfaction.

Patients without a history of pregnancy reported slightly higher mean satisfaction scores than those with previous pregnancies. However, both groups were well represented among those who were “very happy,” indicating that pregnancy history was not a strong determinant of satisfaction.

Patients with medium-sized implants (301-400 cc) achieved the highest mean satisfaction scores, followed by those with smaller implants (≤300 cc). Patients with larger implants (>400 cc) reported slightly lower satisfaction and included those who were “satisfied” or “not happy,” suggesting that moderate implant volumes may be more strongly associated with positive satisfaction outcomes.

Regarding pocket type, DP2 was the most frequently used and had the highest proportion of patients reporting being “very happy.” Patients with DP1, DP3, or other pocket types also reported high satisfaction levels, with no cases of dissatisfaction. These findings suggest that DP2 placement may offer a slight advantage in maintaining higher satisfaction compared with other pocket configurations.

**Table 2:** Patient satisfaction scores

Satisfaction responses	Patient satisfaction score
Very happy	54 (91.5%)
Happy	3 (5.1%)
Not happy	1 (1.7%)
Satisfied	1 (1.7%)

Complication rates

Of the 59 patients, complications were reported in four cases (6.8%). These included one case of bilateral bottoming out, two cases of implant loss due to infection, and one case of unilateral capsular contracture. The majority of patients (55, 93.2%) experienced no postoperative complications. In the case of the patient who developed bilateral bottoming out, the nipple position was at the green line, which would ideally have indicated the need for breast augmentation with a circumvertical mastopexy. In an effort to avoid mastopexy, the senior author opted for augmentation alone. Although the patient later required revision for bottoming out, consisting of lower pole excision and tightening of the soft tissue envelope, she ultimately retained only an IMF scar without periareolar or vertical scars. Despite the revision, this was considered a positive outcome given the patient’s preference to avoid a vertical scar.

**Table 3:** Complication rates

Complications	% of cases
Complications reported	4 (6.8%)
No complications reported	55 (93.2%)

Representative case example

**Figure 2:**
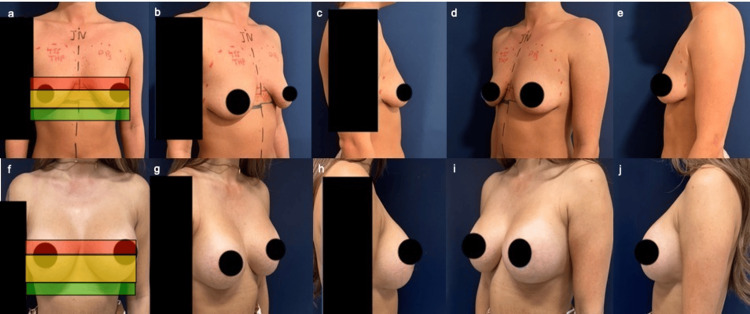
Pre-op photos (top) and post-op photos at six months with nipple above red line (bottom) Top: (a) AP showing nipple in amber zone, (b) right oblique, (c) right lateral, (d) left oblique, (e) left lateral. Bottom: (f) AP, (g) right oblique, (h) right lateral, (i) left oblique, (j) left lateral.

An example of the Traffic Light Planning Algorithm in practice is shown in a 36-year-old woman with early ptosis (amber zone) and inframammary fold (IMF) asymmetry (Figure 2). The patient underwent bilateral breast augmentation using 455 cc overfilled round high-profile Mentor MemoryGel Xtra implants placed in a DP3 pocket. IMF asymmetry was corrected by using the higher right IMF as a reference to level the contralateral side. Postoperative photographs at six months demonstrate good IMF symmetry, indicated by a balanced takeoff of both breasts, with no uplift techniques required and no evidence of bottoming out.

## Discussion

The Traffic Light Planning Algorithm provides a clear and objective framework that helps surgeons navigate the complexity of augmentation-mastopexy planning. By using fixed anatomical landmarks, specifically the IMF and the Per Heden-guided implant-created nipple lift, surgeons can minimize subjective estimations and achieve more reproducible results. This approach aligns with established principles described by Regnault, Tebbetts, and Adams [3-7]. Regnault introduced a clinically useful classification of breast ptosis, while Tebbetts developed a tissue-based decision support tool for breast augmentation, initially known as the TEPID system. This was later refined into the widely used High Five System, which defines patient-specific tissue characteristics to guide decisions regarding implant size, pocket selection, weight, and dimensions. Building on these foundational concepts, our Traffic Light Planning Algorithm objectively defines the degree of ptosis and incorporates the effect of implant-created nipple lift into surgical planning.

Early ptosis presents a significant challenge in implant-only augmentation, often resulting in suboptimal aesthetic outcomes if not managed with either a supportive implant or a minor lift. Overfilled, form-stable implants can offer subtle upper pole support, reducing the need for extensive mastopexy in mild cases. When more advanced ptosis is present, adding mastopexy ensures appropriate nipple elevation and tightening of the skin envelope, helping prevent complications such as the double-bubble deformity. The Traffic Light Planning Algorithm provides a structured guide to identify the threshold, within the amber and green zones, where implant lift alone becomes insufficient and where adjunctive mastopexy may be required to achieve the desired nipple position at the breast apex. In addition, the algorithm offers an objective framework for determining whether a one- or two-stage approach is most appropriate. This may help lower the reported revision rates of 6%-21% associated with single-stage procedures, which are often linked to wound breakdown, pseudoptosis, and capsular contracture [13,14]. Incorporating the degree of excess vertical tissue as a key parameter in this decision also aligns with recommendations by Xue et al., who suggested that a nipple elevation exceeding 4 cm favors a two-stage approach [14].

With regard to the use of MemoryGel Xtra implants for early ptosis, our results indicate high patient satisfaction and a low complication rate. These findings are consistent with the GLOW Clinical Trial, which evaluated the safety and effectiveness of MemoryGel Xtra implants across multiple settings, including primary augmentation, revisional augmentation, primary reconstruction, and revisional reconstruction, involving 287 participants [9]. In that study, complication rates for primary augmentation were low, with 1.5% for implant-related reoperations, 2.3% for explantations, and 1.5% for Baker Grade III or IV capsular contracture. Revisional augmentations showed slightly higher rates, while primary reconstruction reported 12.0% for reoperation, 12.3% for explantation, and 7.3% for capsular contracture. Across all groups, there were no cases of infection, malposition, or displacement, and participants reported significant improvements in breast satisfaction, psychosocial well-being, and sexual well-being one year postoperatively. Although our study specifically focuses on early breast ptosis, a subgroup that has not been extensively studied, our positive outcomes align with the broader evidence supporting the safety and performance of these implants.

A major advantage of MemoryGel Xtra implants lies in their optimized gel fill and shell design, which helps reduce complications such as wrinkling, rippling, and capsular contracture. The GLOW Clinical Trial reported low complication rates at three years: reoperation (1.5%), explantation (2.3%), and capsular contracture (1.5%) [9]. In our cohort, the overall complication rate was similarly low (6.8%), with isolated cases of bilateral bottoming out, implant loss due to infection, and unilateral capsular contracture. These findings support the use of MemoryGel Xtra implants as a reliable option for managing early breast ptosis, where additional internal support helps prevent implant descent.

The enhanced gel fill of MemoryGel Xtra implants provides a subtle “internal lift,” improving projection and minimizing rippling, features that are particularly useful in mild ptosis where mastopexy is not indicated. Benyaminpour and Shalom also noted that increased gel fill reduces wrinkling and rippling, contributing to improved implant longevity [15]. In early breast ptosis, these attributes help maintain upper pole fullness and reduce postoperative complications.

Patient satisfaction in our study was notably high, with 91.5% of patients reporting being “very happy.” This mirrors the GLOW trial’s patient-reported outcomes, where BREAST-Q assessments demonstrated significant improvements in breast satisfaction, psychosocial well-being, and sexual well-being at one year. Collectively, these findings emphasize both the aesthetic and psychological benefits of MemoryGel Xtra implants in optimizing outcomes for patients with early breast ptosis.

Strengths and limitations

Although our approach is based on extensive clinical experience and draws from established anatomical concepts, it is not without limitations. The algorithm does not replace surgical judgment and may require adaptation for individual patient anatomy, tissue quality, and aesthetic goals. It has been developed and refined by a single practice over many years; broader validation, comparative studies with other planning systems, and long-term follow-up data would strengthen its evidence base.

For new surgeons, the algorithm’s structured approach fosters confidence and consistency. By following objective measures rather than subjective assessments, it simplifies patient counseling and expectation setting. Our series indicate a high patient satisfaction level of 93% and a low revision rate of 2% for augmentation alone and 6% for augmentation mastopexy, which is comparable to other series [16,17]. Patients benefit from understanding the rationale behind recommended procedures, promoting realistic expectations and higher satisfaction.

## Conclusions

The Traffic Light Planning Algorithm offers surgeons a structured, objective framework for planning breast augmentation with or without mastopexy. By anchoring decisions in anatomical landmarks and clearly defined zones of ptosis, this approach supports individualized surgical planning, reduces complications, and enhances the predictability of outcomes. The Traffic Light Planning Algorithm builds on work by others, providing a simple visual system, which is particularly useful in deciding about a one- or two-staged procedure. While future research may further validate its efficacy and reproducibility, the algorithm provides a valuable roadmap, particularly for those new to aesthetic breast surgery, leading to improved patient satisfaction and lasting results.

MemoryGel Xtra implants are an effective option for early breast ptosis, achieving high patient satisfaction with a low complication profile. Their enhanced projection and stability make them particularly suitable for patients with moderate soft tissue laxity. Further research with larger cohorts and extended follow-up is required to validate these findings and refine best practices for implant-based management of early breast ptosis.

## References

[1] Triana L, Palacios Huatuco RM, Campilgio G, Liscano E (2024). Trends in surgical and nonsurgical aesthetic procedures: a 14-year analysis of the International Society of Aesthetic Plastic Surgery-ISAPS. Aesthetic Plast Surg.

[2] Stevens WG, Calobrace MB, Harrington J, Alizadeh K, Zeidler KR, d'Incelli RC (2016). Nine-year core study data for Sientra's FDA-approved round and shaped implants with high-strength cohesive silicone gel. Aesthet Surg J.

[3] Regnault P (1976). Breast ptosis. Definition and treatment. Clin Plast Surg.

[4] Tebbetts JB (2006). Achieving a zero percent reoperation rate at 3 years in a 50-consecutive-case augmentation mammaplasty premarket approval study. Plast Reconstr Surg.

[5] Adams WP Jr (2008). The process of breast augmentation: four sequential steps for optimizing outcomes for patients. Plast Reconstr Surg.

[6] Tebbetts JB, Adams WP (2006). Five critical decisions in breast augmentation using five measurements in 5 minutes: the high five decision support process. Plast Reconstructivive Surgery.

[7] Tebbetts JB (2002). Breast implant selection based on patient tissue characteristics and dynamics: the TEPID approach. Plast Reconstr Surg.

[8] Hedén P (2009). Mastopexy augmentation with form stable breast implants. Clin Plast Surg.

[9] Alderman A, Caplin D, Hammond DC, Keane A, Turetzky J, Kane WJ (2023). Clinical results of mentor MemoryGel Xtra breast implants from the GLOW clinical trial. Aesthetic Surgery Journal.

[10] Jebb AT, Ng V, Tay L (2021). A review of key Likert scale development advances: 1995-2019. Front Psychol.

[11] Arora N, Patel R, Sohi G, Merchant S, Martou G (2023). A scoping review of the application of BREAST-Q in surgical research. JPRAS Open.

[12] (2024). The R Project for Statistical Computing. https://www.R-project.org/.

[13] Khavanin N, Jordan SW, Rambachan A, Kim JY (2014). A systematic review of single-stage augmentation-mastopexy. Plast Reconstr Surg.

[14] Xue AS, Dayan E, Rohrich RJ (2020). Achieving predictability in augmentation mastopexy: an update. Plast Reconstr Surg Glob Open.

[15] Benyaminpour S, Shalom M (2024). Optimizing breast implant outcomes: MemoryGel Xtra implants and future research directions. Aesthetic Surgery Journal.

[16] Spear SL, Dayan JH, West J (2009). The anatomy of revisions after primary breast augmentation: one surgeon’s perspective. Clin Plast Surg.

[17] Spear SL, Boehmler JH, Clemens MW (2006). Augmentation/mastopexy: a 3-year review of a single surgeon’s practice. Plast Reconstr Surg.

